# A Randomized Controlled Exploratory Evaluation of Standardized Ayurvedic Formulations in Symptomatic Osteoarthritis Knees: A Government of India NMITLI Project

**DOI:** 10.1155/2011/724291

**Published:** 2010-10-11

**Authors:** Arvind Chopra, Manjit Saluja, Girish Tillu, Anuradha Venugopalan, Sanjeev Sarmukaddam, Ashwini Kumar Raut, Lata Bichile, Gumdal Narsimulu, Rohini Handa, Bhushan Patwardhan

**Affiliations:** ^1^Centre for Rheumatic Diseases (CRD), Hermes Elegance,1988 Convent Street, Camp, Pune 411001, India; ^2^Interdisciplinary School of Health Sciences (SHS), University of Pune, Pune 411007, India; ^3^Rheumatology Departement, KEM Hospital and GS Medical College and SPARC, Parel, Mumbai 400012, India; ^4^Rheumatology Departement, Nizam Institute of Medical Sciences (NIMS), Panjagutta, Hyderabad 500082, India; ^5^Rheumatology Departement, All India Institute of Medical Sciences (AIIMS), Ansari Nagar, New Delhi 110029, India

## Abstract

The multidisciplinary “New Millennium Indian Technology Leadership Initiative” Arthritis Project was undertaken to validate Ayurvedic medicines. Herbal formulations in popular use were selected by expert consensus and standardized using modern tools. Our clinical strategy evolved from simple exploratory evaluations to better powered statistically designed drug trials. The results of the first drug trial are presented here. Five oral formulations (coded A, B, C, D and E), with a common base of *Zingiber officinale* and *Tinospora cordifolia* with a maximum of four plant extracts, were evaluated; with placebo and glucosamine as controls. 245 patients suffering from symptomatic OA knees were randomized into seven arms (35 patients per arm) of a double blind, parallel efficacy, multicentric trial of sixteen weeks duration. The groups matched well at baseline. There were no differences for patient withdrawals (17.5%) or adverse events (AE) of mild nature. Intention-to-treat efficacy analysis, demonstrated no significant differences (*P* < .05) for pain (weight bearing) and WOMAC questionnaire (knee function); placebo response was high. Based on better pain relief, significant (*P* < .05) least analgesic consumption and improved knee status, “C” formulation was selected for further development. Controlled exploratory drug trials with multiple treatment arms may be used to economically evaluate several candidate standardized formulations.

## 1. Introduction

Ayurveda, a holistic ethnic medicinal system [1],is in practice in India and Sri Lanka since the prebiblical era [2] and has contributed to discovery and development of natural product drug [3]. The traditional system advocates predominantly life style (includes diet) approach to good health and disease prevention. When treating a disease, herbal mineral formulations are added to the latter approach after assessing an individual's constitution traits (called *prakriti *in Ayurveda) [2]. Undoubtedly, the traditional approach is predominantly individual and patient centric conforming to the basic principle of “no two individuals are alike.” Unlike a reductionist approach to strike the bull's eye (modern medicine), Ayurvedic treatment attempts to correct several factors implicated in causing the disease (called *samprapti *in Ayurveda). The system advocates a “soil more important than the seed” approach. Though therapeutic managements may differ, Ayurvedic physicians often use certain treatment modalities (e.g., the well-known detoxification technique called *panchkarma* in Ayurveda) and drugs in a uniform manner to treat different disorders. Chronic arthritis, irrespective of its etiology or clinical profile, is also one such disorder. Therefore, it may be reasonable to evaluate Ayurvedic drugs per se for a more general therapeutic use. But this would entail systematic validation using contemporary scientific methods and evidence [4]. Ayurveda has an elaborate disease classification system. In the case of classification of rheumatic disorders [5], we have reported some similarity between modern medicine and Ayurveda. Recently, a concept of “golden triangle” between modern science, modern medicine, and traditional medicine was proposed to promote integrated research and development [6].

Ayurvedic medicinal plants have demonstrated remarkable biological effects,especially those of anti-inflammatory and immunomodulatory activities [7–10], that are relevant and potentially useful to treatment of chronic musculoskeletal disorders. We have carried out several controlled drug trials to demonstrate efficacy and safety of standardized Ayurvedic drugs containing several plants mentioned in this paper, for treatment of osteoarthritis (OA) and rheumatoid arthritis [11–14].

An Ayurvedic segment of the “New Millennium Indian Technology Leadership Initiative” (NMITLI), a multidisciplinary national project, fully sponsored by the Government of India, was launched in 2002. The principal aim was to validate standardized Ayurvedic medicines for global use. Arthritis, diabetes, and hepatitis were selected as the target disorders. Experienced Ayurvedic physicians were involved at every step. The candidate drugs were assessed essentially using the “reverse pharmacology” approach. This approach gets credence from the historical and experiential long-term safe use of Ayurvedic medicines over several hundred years wherein validation of clinical use precedes or goes hand in hand with the pharmacological and other relevant mechanism of action studies. 

**Figure 1:**
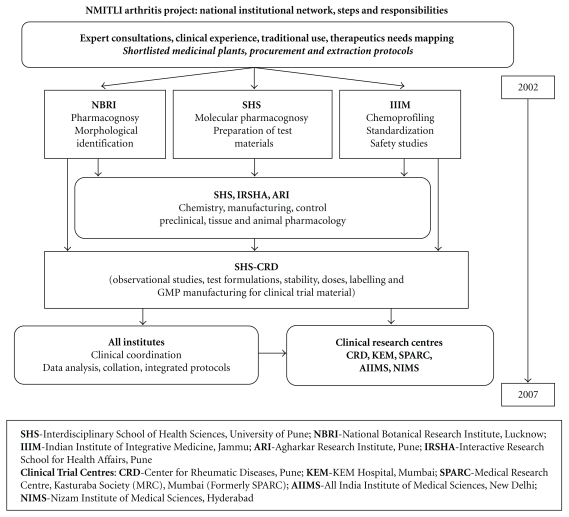
NMITLI Arthritis Project: National Institutional Network, steps and responsibilities. **ISHS** Interdisciplinary School of Health Sciences, University of Pune; **NBRI** National Botanical Research Institute, Lucknow; **IIIM** Indian Institute of Integrated Medicine, Jammu; **ARI** Agharkar Research Institute, Pune; IRSHA Interactive Research School for Health Affairs, Pune; **Clinical Trial Centres**: **CRD** Center for Rheumatic Diseases, Pune; **KEM** KEM Hospital, Mumbai; **SPARC** Medical Research Centre, Kasturaba Society (MRC), Mumbai (Formerly SPARC); **AIIMS** All India Institute of Medical Sciences, New Delhi; **NIMS** Nizam Institute of Medical Sciences, Hyderabad; **Industry**: **NR** Natural Remedies, Bangalore; Zandu Pharmaceutical Works, Mumbai; Arya Vaidya Pharmacy, Coimbatore; Arya Vaidya Shala, Kottakal; Dabur India Ltd, New Delhi; Nicolas Piramal India Ltd. Mumbai.

We selected OA knees as the target disorder. The arthritis team involved a network of seventeen national research institutions, hospitals with modern medicine, and pharmaceutical industries with a delegation of specific responsibilities (Figure 1). To begin with, Ayurveda and related discipline experts interacted in brain storming sessions to determine diagnostic and other critical protocol components (outcome measures and response criteria, in particular) and shortlisted medicinal plants with a potential to treat OA knees. During the selection process, due emphasis was given to the most popular traditional and proprietary formulations, in Ayurvedic clinical practice in India. Though authoritative literature reviews, scientific evidence and expert opinions were given due importance, the team adopted a consensual approach to finalize several key selections and procedures and settle contentious operative issues, if any. Though based on traditional principles of Ayurvedic pharmacology and pharmaceutics, formulations were developed and standardized using modern science methods. Safety data from animal studies was submitted to Institutional Ethics Committee to obtain clearance prior to the clinical drug trials. In a step-wise clinical approach (from exploratory to statistically designed large sample size controlled drug trials), the formulations were systematically evaluated (for efficacy and safety) with a view to identify and validate the best formulation. We present the results of the first of a series of planned drug trial evaluations completed under the auspices of the NMITLI project.

## 2. Patients and Methods

This trial was conducted at the Centre for Rheumatic Diseases (CRD, Pune), Nizam Institute of Medical Sciences (NIMS, Hyderabad), KEM Hospital and GS Medical College (KEM, Mumbai), and Swami Prakashananda Ayurveda Research Centre (SPARC, Mumbai). The protocol was duly approved by the institutional ethics committees of the respective centers. Prior to signing the informed consent, patients were properly informed and counseled with a detailed multilingual brochure.

### 2.1. Design

This was a randomized, double blind, placebo and oral glucosamine controlled, parallel efficacy, multicentre drug trial study of 16 weeks duration using seven intervention treatment arms. The trial was essentially exploratory in nature and not statistically designed for sample size nor powered (for a low Type II error). Patients were examined by modern medicine physicians (in particular rheumatologists) and Ayurvedic physicians.

### 2.2. Ayurvedic Formulations (Tables 1 and 2)

#### 2.2.1. Selection

Classic Ayurvedic text and medicinal plant experimental data that is published was referred by the experts to select the most favored candidate plants and create a medicinal plant inventory. All the selected plants were well known and documented in classic literature [15] and described to possess analgesic, anti-inflammatory, and immunomodulation properties and also to promote positive health. Herbal and mineral formulations possessing such a combination of preventive and therapeutic effects are described as *Rasayana* (immunomodulators and facilitating regeneration) in Ayurveda and have been widely used by Ayurvedic physicians since ancient times to promote health and treat immunoinflammatory and degenerative disorders. Using systems biology approach, the medicinal properties of selected plants were matched with the desired clinical efficacy targets (both from an Ayurvedic and modern medicine view point) in an activity matrix to select the formulations. The targets included analgesia, anti-inflammatory, chondroprotection, soft tissue healing, antiosteoporosis, immunomodulation, antilipogenesis, anabolic effect, and anti-oxidative stress, in order to promote digestion and normal gut function. One of the fundamental objectives of Ayurvedic antiarthritis drugs and regimen is to improve digestion and metabolism [6].

#### 2.2.2. Test Materials

All the raw materials were procured from known authorized suppliers who provided passport data in the form of history data sheets. All the botanical drugs including Shunthi (*Zingiber officinale*), Guduchi (*Tinospora cordifolia*), Amalaki (*Emblica officinale*), Ashwagandha (*Withania somnifera*), and Gokshur (*Tribulus terrestris*) were identified and authenticated by Ayurvedic experts and medicine plant botanists. Routine pharmacognosy and chemo profiling with at least one phytochemical marker (on HPLC technique) was also completed (Table 1) by National Botanical Research Institute, Lucknow, India. Voucher samples (Table 1) of all botanical materials were deposited in the official herbarium of Agharkar Research Institute, Pune. Five Ayurvedic formulations (Table 2), coded as A, B, C, D, and E, were used as investigational drugs. Batch samples of raw material were properly labeled, sealed and stored in SHS, Pune, for any future reference or investigation. After ensuring quality control standards, the raw material was processed for extraction.

#### 2.2.3. Development, Standardization, and Manufacture

Based on traditional knowledge and popular use in clinical practice, Shunthi and Guduchi were first selected and optimized to create a platform of base formulation. Synergistic ingredients from the plant inventory were added to create variants (Tables 1 and 2). Aqueous extracts were prepared using traditional Ayurvedic procedures [16]. Organic solvents were not used. The precise quantity of each plant extract ingredient in the formulation was guided by the standard Ayurvedic teaching text [17] and finally fixed by Ayurvedic experts. 

Overall, the entire process comprised of several predefined milestone deliverables and suitable tests starting from creating passport data of raw material, identifying botanical material (pharmacognosy), to ensuring chemical quality (Spectroscopic and Chromatographic), molecular (DNA Fingerprinting) standardization, stability, and pharmacopoeia standards of finished product. Necessary documentation was maintained for review, records, and regulatory needs.

The drug trial test material (Ayurvedic formulations, placebo, and glucosamine) was manufactured as capsules with uniform weight (approx. 500 mg), similar appearance, and smell. Only permitted pharmaceutical grade excipients were used. Pharmaceutical grade charcoal, and synthetic ginger essence were used to give uniform appearance and smell to placebo and glucosamine. The standards were consistent with the guidelines for botanical drugs on GMP (Good Manufacturing Practices) and CMC (Chemistry Manufacture and Control) provided by the US FDA to the industry [18].The excipients included pharmaceutical grade maize starch, talc, charcoal and synthetic ginger essence. The placebo capsules were filled with maize starch and were identical in size, shape, color, and odor to those of the formulations. Tests were also carried out for microbial load, heavy metals, pesticide residues, and aflatoxins as per standard norms.

#### 2.2.4. Safety and Activity

Animal toxicity studies were carried out at Agharkar Research Institute, Pune, as per OECD guidelines Serial Number 423 [19]. The acute and subacute studies were completed prior to the initiation of clinical trials while the chronic toxicity studies were completed later. None of the animal studies demonstrated any obvious toxicity. 

In-house data was generated, both in animal and lab experiments, to support some of the putative properties and actions of the selected plants. Moderate analgesic and anti-inflammatory activities were demonstrated in acute and chronic standard animal pharmacology models. As compared to single drugs, multiple plant formulations exhibited better efficacy to support the contention of a synergistic clinical activity expected from the trial formulations (data not shown). Human cartilage (procured during knee surgery) was cultured in an artificial medium to set up human OA explants cartilage model which demonstrated beneficial effects of the candidate plant extracts and formulations on some of the critical cellular processes (proteoglycan release, nitric oxide release, aggrecan release, and hyaluronidase inhibition ) to further support their clinical use [20–22]. The selection process of candidate medicinal plants in this project appeared to be vindicated by several experimental studies.

### 2.3. Patient Selection

Patients suffering from symptomatic OA knees were screened for eligibility as per the protocol in rheumatology outpatient clinics of participating medical institutions and free-of-cost knee arthritis camps carried out in community settings. The camp methodology was often used by CRD, Pune, to meet the large enrollment target of 147 eligible patients within the study time frame. Volunteer patients signed informed consent to enroll in the trial.

#### 2.3.1. Inclusion Criteria

Patients of either gender belonging to the 40 to 70 years age group; diagnosis of OA knees based on typical history, clinical examination findings and classical radiological findings, and fulfilling the ACR classification criteria [23] except that the lower age limit was reduced to 40 years; pain visual analogue score (VAS) ≥4 cms in one or both the knees while performing a weight bearing activity (e.g., walking, standing, climbing staircase) during the preceding 24 hours; patients who were ambulant and required analgesic and/or NSAID (nonsteroidal anti-inflammatory drug, e.g., ibuprofen) for pain relief and/or not satisfied with ongoing analgesic drugs and seeking a change.

#### 2.3.2. Exclusion Criteria

Women who were pregnant, lactating, and having child bearing potential and not following adequate contraceptive measures; patients with known contraindication to any of the investigational products and medicinal plants; those who had nondegenerative joint diseases or other joint diseases which would interfere with the evaluation of OA; patients with severe disabling arthritis and/or the patient was who incapacitated and bedridden; those who had history of intra-articular knee injection (in particular corticosteroids and hyaluronon equivalents) within the month preceding the study; those who were ongoing treatment with anticoagulants, hydantoin, lithium, steroids, methotrexate, and colchicine; those with history of active peptic ulcer at any time in the preceding six months or bleeding ulcer at any time in the past; those with evidence of severe unstable renal, hepatic, hemopoietic, and cardiac disorder as revealed by history and/or investigations; those with history of having received any investigational drug in the previous one month; patients taking antipyretics, analgesics, tranquilizers, hypnotics, excessive alcohol, or any other drug which would interfere with pain perception and need for other drug therapy for OA, except paracetamol (allowed as a rescue drug during the study period); those unwilling to come for regular follow-up for the entire duration of the study and any patients considered not eligible according to the investigator's discretion.

#### 2.3.3. Wash-Out Period

The patients using NSAID prior to enrolment were entered into a supervised wash-out period. Standard recommendations (based on plasma half life) were followed if the NSAID was known and a maximum of five days wash-out was carried out if the name of analgesic/NSAID used by the patient was unknown. All other pain relieving medications were discontinued but oral paracetamol (500 mg tablet taken 3 to 4 times in a day) was permitted as a rescue medication on need basis. However, if the pain became intolerable, the wash-out phase was terminated prematurely and the patient entered the trial intervention phase.

### 2.4. Randomization

Eligible patients were enrolled on “first come first serve” basis and assigned a treatment arm based on a randomization schedule generated by standard software under the supervision of a senior investigator (B. Patwardhan) who was not actively associated with the actual clinical trials. Patients were randomized in a 1 : 1 ratio in any of the seven treatment groups. Each centre was assigned blocks in multiples of seven according to the predetermined target of “number of patients to be enrolled.” 

### 2.5. Clinical Evaluation [24]

End point evaluation visits were made at baseline and at weeks 2, 4, 8, 12, and 16. Active pain (on weight bearing) and WOMAC index were considered the primary efficacy variables and recorded at every visit.

#### 2.5.1. Active-Pain VAS

Patients recorded maximum pain experienced in both the knees separately on a horizontal 10 cms VAS (anchored at 0 for absent pain and 10 for maximum pain) during weight bearing activity.

#### 2.5.2. Western Ontario and McMaster University's OA Index Version LK3 (WOMAC) [25]

A validated and modified version of WOMAC questionnaire for Indian use [26] was used to assess pain, stiffness, and functional ability in the knees. The version was further translated into several Indian regional languages by CRD, Pune, and appropriately retested for content and comprehension (using back translations and in small independent patient groups) prior to the actual trial. The pain and stiffness domains in the Indian version are unchanged from the original version. However, several questions from the physical function “difficulty” domain have been removed and replaced by those relevant to Indian customs and traditions (especially those who squat and sit cross legged). Each of the questions in the pain (5 questions), stiffness (2 questions), and physical function difficulty (17 questions) domain was scored by the patient in a face to face interview conducted by a trained trial paramedic into one of the categorical answers (none = 0, mild = 1, moderate = 2, severe = 3, extreme = 4). The score of all the answers was summed (24 questions with a maximum score of 96) up. 

Secondary efficacy variables (clinical and laboratory) were also recorded and included in pain VAS on rest, and in walking time (time taken to walk a 50 feet ground level distance), physician and patient global assessment (graded from asymptomatic to a very severe category) of disease, in patient's graded assessment of drug tolerability, change in the knee status on completion of the study as assessed by the patient (worse = 1, no change = 2, mild improvement = 3, moderate improvement = 4, marked improvement = 5 ), and paracetamol consumption. A fixed amount of paracetamol tablets (500 mg each tablet) were provided at each visit according to a predetermined scale contained in the protocol. During the predetermined follow up visit, the number of tablets that were not consumed were withdrawn and recorded. As per the scale, a reducing amount of paracetamol was issued at each predetermined follow-up visit. Laboratory variables included several serum cytokine and hyaluronic acid assays. 

An Ayurvedic CRF was also completed by an Ayurvedic physician (data not presented).

### 2.6. Treatment

The prescribed dose was two capsules twice a day to be taken with plain water after meals (lunch and dinner). The daily dose of glucosamine sulfate was 1000 mg (250 mg/capsule). Concomitant medication, if ongoing and fixed over time, for concurrent illnesses was permitted. Patients could continue their regular exercise and/or physiotherapy program begun prior to the current trial but were discouraged from starting any new activity during the trial duration. Physical therapy and local applications of pain relieving ointments/gels for OA knees were not permitted. Patients were not allowed to seek therapy from any other alternative medicinal system (such as homeopathy, acupuncture, and acupressure).

### 2.7. Laboratory Investigations

The focus of investigations was on safety rather than any efficacy parameter. Routine laboratory workup was done at baseline and on completion of the study. Routine workup included hematology (total and differential white blood cell count, platelet count, erythrocyte sedimentation rate by Westergren method), biochemistry (blood sugar—Fasting), blood urea, serum creatinine, serum calcium, serum uric acid, serum bilirubin, total serum proteins/albumin/globulin, serum amino-transferases), rheumatoid factor assay, and urinalysis. Routine EKGs were taken for all patients at entry. X-rays of knees were taken to confirm diagnosis. However, as per the protocol, some special laboratory tests such as serum IL-1, IL-6, and TNF-*α* and Hyaluronic acid were carried out more from a research point of view.

### 2.8. Adverse Events

Patients were specifically questioned as per a predetermined list of common symptoms (anorexia, nausea, vomiting, diarrhea, constipation, dysuria, skin rash, giddiness, oral mucous ulcers, dyspepsia, and abdominal discomfort and pain) based on our experiences in clinical practice. Patients were also encouraged to volunteer information that they considered to be adverse events (AE) or a side effect (SE). Severe and life-threatening AE were to be investigated, treated, and notified as per protocol and GCP guidelines. The investigator recorded opinions on the causality/relevance of AE/SE in each case.

### 2.9. Withdrawals

Patients could withdraw voluntarily or at the discretion of the Investigator. Patients were not replaced and the new patient who was enrolled was allotted the next consecutive randomization number. Efforts were made in each case to identify the reason for a failed follow-up visit and/or withdrawal.

### 2.10. Blinding, Monitoring, and Trial Database

Blinded coded trial material (investigational products) and randomization schedules were provided by SHS. As the coordinating center, CRD organized pretrial sessions for protocol discussion and standardization of clinical procedures. The clinical coordinator (M. Saluja) from CRD visited other trial sites at regular intervals to check trial progress and documents as per the GCP guidelines. An independent referee was also designated by the CSIR to visit all the trial sites and carry out a trial audit. After relevant checks, a copy of coded trial data was submitted to CSIR (Sponsor and Monitor) and SHS. The trial data was then decoded under supervision of S. Sarmukaddam (CRD) and B. Patwardhan (SHS) and kept locked (password protected). One copy of the locked database was also submitted to CSIR and SHS prior to carrying out statistical analysis. Trial data entry was supervised by M. Saluja. Statistical analysis was carried out by S. Sarmukaddam and his colleagues.

### 2.11. Statistical Analysis

At baseline,the qualifying knee/knees were identified as the signal joint/joints for subsequent primary efficacy variable analysis. However,each knee was examined and recorded separately at each visit. Sample size was not calculated as per any statistical method and no assumptions were made regarding “effect size.” At least 30 patients were required to complete the trial in each arm. Efficacy was assessed by an intention-to-treat analysis with the “last observation carried forward.” Significance was ascertained at *P* < .05. Confidence intervals (95%) were calculated for all means (of variables) and mean change over time. We compared the mean change from baseline to completion in each of the intervention arm using ANOVA. Within group efficacy was evaluated by one sample Student's *t*-test. Chi-square test was done for all categorical outcomes including the side-effects/adverse events. Standard statistical software program SPSS version 12.5 was used.

## 3. Observations and Results

### 3.1. At Baseline, the Intervention Arms Were Well Matched

**Figure 2:**
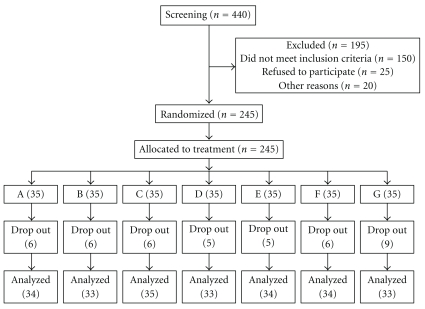
Flow of participants in a seven arm (Five Ayurvedic formulations coded A, B, C, D, and E; oral glucosamine coded G, oral placebo coded F) exploratory drug trial study of patients with symptomatic osteoarthritis knees (see text for details).

245 patients (176 females among them) were randomized into seven arms. The flow of participants in the study is shown in Figure 2. This project trial was begun on 26 April, 2002 (first investigator meeting), and the statistical data analysis report was submitted on 31 March, 2005, over a total period of twenty three months. 

The groups were well matched for several baseline demographic and other disease measures as shown in Table 3. 

Concomitant disorders recorded in the study were hypertension (50 patients), diabetes (16 patients), hyperlipidemia (3 patients), ischemic heart disease (3 patients), bronchial asthma (1patient), and acid peptic disorders (4 patients); the numbers of patients are shown in parenthesis.

### 3.2. Eighteen Percent Patients Withdrew from the Study but None Due to Serious Adverse Events

Forty three patients withdrew from the study (as per arm: 6 in A, 6 in B, 6 in C, 5 in D, 5 in E, 6 in placebo, and 9 in the glucosamine arm). There were no obvious differences between the reasons for withdrawal in any of the groups. However, the reasons were loss to follow-up (15 patients), noncompliance (4 patients), change in location (5 patients), poor efficacy (8 patients), and frequent abdominal upsets/diarrhea (5 patients); not more than 2 patients ever withdrew from any of the intervention groups for any of the cited reasons. None of the patients who withdrew, required hospitalization. Though we did not grade any AE severe, five patients (including 2 each from placebo and glucosamine arms) stopped medication due to frequent abdominal upsets and withdrew from the study.

### 3.3. Only Mild Adverse Events Were Reported with Least Incidence in the C Treatment Arm

None of the patients in the current study reported serious AE. All AE were mild and were treated symptomatically. None of the patients with AE required invasive intervention or an imaging study. The common AE/SE reported were epigastric discomfort, vague diffuse dull pain in abdomen, anorexia, nausea, diarrhea, constipation, and episodic skin rash (mostly nonitchy). Overall, a lesser frequency of AE seemed to be reported in the “C” arm as compared to other Ayurvedic intervention arms, glucosamine, and placebo.

### 3.4. Amongst All the Groups, “C” Formulation Showed the Best Response in Reducing Pain

**Figure 3:**
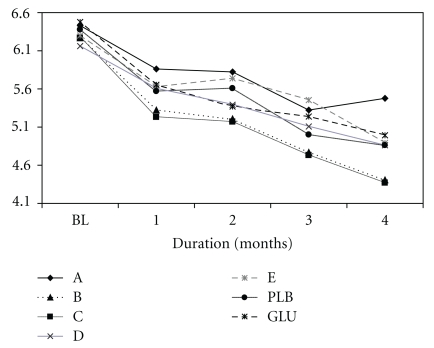
Change in painVAS overtime (months) by treatment group.

There was no statistically significant difference (at *P* < .05) in the mean change from baseline to completion in active-pain VAS and WOMAC index between the groups (Table 4). However, the maximum reduction in pain was demonstrated by the “C” formulation (Table 4) and this was further demonstrated at every follow-up evaluation (Figure 3). The latter improvement was further supported by an unequivocal demonstration of lesser consumption (*P* < .05) of a number of oral paracetamol tablets, both during the total study period and the final four weeks (Table 4). We also analyzed the proportion of patients showing >60% reduction in the active-pain VAS from baseline to completion and found that the “C” arm (12 patients) was ahead of the placebo (8 patients) and glucosamine (4 patients) groups. Analysis of the data on knee status (Section 2) on completion of the study demonstrated the best improvement in the “C” arm; a RIDIT (relative to identified distribution) analysis (Table 5) showed that the “C” formulation was definitely better than the placebo (*P* = .03) or glucosamine (*P* = .09). The latter was interpreted to mean that more than half of the time, a randomly selected subject from “C” arm was likely to have a better knee status as compared to a randomly selected individual from any of the reference groups (placebo and glucosamine). Though marginal and modest, the “C” arm consistently showed a better numerical response for several other secondary efficacy measures ( Section 2)as compared to placebo,glucosamine and several other Ayurvedic drug intervention (data not shown).

The placebo response was found to be unexpectedly strong, especially with reference to the WOMAC result (Table 4).

### 3.5. Routine Laboratory Parameters of Safety Remained within Normal Range

There were no statistically significant differences between the groups for routine hematological, metabolic, renal, and hepatic parameters (data not shown). Though modest and not significant, we observed the best mean change (drop) in serum hyaluronic acid from baseline to completion in the “C” arm (data not shown). We also measured selected serum cytokines (IL-6, IL-1, and TNF-*α*) in patients belonging to B, C, glucosamine, and placebo arms, both at baseline and on completion, but could not find a meaningful change or a consistent trend favoring any group (data not shown).

## 4. Discussion

**Figure 4:**
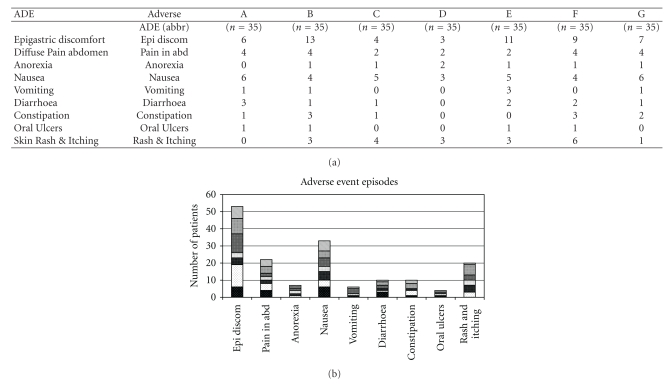
Number of adverse event episodes in patients (*n* = 245) by treatment group: Ayurvedic arms code (A–E), glucosamine (GLU), and placebo (PLB).

In this controlled drug trialstudy comparing several Ayurvedic formulations (with a common Z. *officinale-T. cordifolia *base) with glucosamine and placebo in the treatment of symptomatic OA knee, we demonstrated a better clinical efficacy and safe use of “C” Ayurvedic formulation, albeit not statistically significant. This was an exploratory study intended to identify the best formulations based on positive clinical trends for further development. Importantly, the “C” formulation reduced pain (Table 5 and Figure 3) and requirement of rescue analgesic (Table 4) and improved the patient centered knee status on completion of study (Table 5). The decrease in the mean pain VAS from 6.3 cms at baseline to 4.4 cms on completion in the “C” arm was clinically meaningful. Though, the incidence of AE and rate of withdrawal from study did not discriminate between the intervention arms (Figure 4), the “C” formulation recorded a comparatively lesser frequency of AE related to abdominal symptoms. 

Our mandate in this initial phase of the NMITLI arthritis project was to identify the most promising formulation for development and validation (a subsequent phase of the project). Accordingly, despite several logistic challenges and an unprecedented trial, we designed a seven arm controlled study of sixteen weeks duration to evaluate five Ayurvedic formulations for their primary pain relief effect. We included both oral glucosamine and placebo as controls. A large number of patients were screened (Figure 2), mostly in a community setting, to select patients of OA with moderately severe knee pain (Table 3). At least 30 patients in each intervention arm completed the study. This trial was not statistically designed nor adequately powered (in order to reduce the chances of a false negative trial result). The clinical trial phase (first patient screened to last patient completed) was completed in about 15 months. We were expected to carry out speedy and economical drug trial evaluations. Though not intended, we now find that the current trial satisfies several of the recently developed recommendations for reporting of trials of herbal interventions [27]. 

We have earlier reported a strong placebo response in Ayurvedic drug trials [11–14] and speculated several causes related to challenging socioeconomics and cultural beliefs in our community. However, the phenomenon of “regression to mean” is well known to masquerade as placebo response. Indians have faith and high expectations from Ayurveda. Improvement in the WOMAC questionnaire (pain domain) is reported to be consistent with the pain relief recorded on VAS [28] but this did not appear to be the case in the current study (Table 4). WOMAC assesses the functional ability of the knees. Perhaps the duration of the current drug trial study was too short to evaluate a meaningful change in WOMAC. Also, WOMAC response in the current study was recorded in one of the six regional languages (English, Hindi, Marathi, Telugu, Gujarati, Punjabi, and Urdu) and probably there was a lack of sufficient standardization between various language versions and paramedic investigators. We carried out a separate analysis of data from each of the study centers but did not find any systematic bias (data not shown). 

Though we chose Ayurvedic formulations which are popularly used in clinical practice, we are aware that the therapy in Ayurveda is holistic and much more complex. Validation of the latter traditional approach using a randomized controlled drug trial may not be the most appropriate method [29]. Since our study aimed at developing standardized formulation for general global use, we adopted a diagnosis centric treatment approach similar to what is practiced in modern medicine. Standardization of Ayurvedic drugs is an extremely important and challenging step in the overall clinical validation process and should not deter investigators to carry out clinical validation studies such as the current report. The current Ayurvedic formulations contained relatively lesser ingredients. The current clinical trial was supported by several experts and accredited national institutions (Figure 1) to carry out the arduous task of standardization and other paraclinical studies including those of animal toxicity which may not be feasible in a routine pharmaceutical setting. Some collateral work from our NMITLI project regarding the likely mechanism of action of the selected plant formulation ingredients has been recently published [21, 22].

We selected OA as the target disorder because it is a common cause of chronic pains, disability, and poor quality of life in the community [30]. Except for physiotherapy and exercises, the therapy options for OA in modern medicine are grossly limited to providing symptomatic relief using analgesic, including nonsteroidal anti-inflammatory drugs (NSAID), or a joint replacement in the end stage situation. Patients often self-medicate and consume analgesics and NSAID for prolonged periods and run a risk of suffering from life-threatening drug toxicity (especially that of gastrointestinal, renal, and cardiovascular system). Effective management of OA would need drugs to repair and strengthen cartilage and prevent future damage. We chose glucosamine in the current study because it is extensively used to treat OA worldwide and has been demonstrated to provide both symptomatic pain relief, improve quality of life, and reduce cartilage damage [31]. But several other well-designed studies have challenged the therapeutic role of glucosamine [32, 33]. The large scale NIH trial [32] demonstrated limited use of oral glucosamine in the treatment of OA. The outcome with glucosamine in the current study (Tables 4 and 5, Figure 3) was rather disappointing but this might have been related to the use of a dose that is currently considered insufficient [34] but was popularly prescribed in our scenario at the time of designing this study. 

 Although envisaged since long, there is a void in the development of chondroprotective drugs. An ideal drug would be one that provides pain relief and chondroprotection. We believe that Ayurvedic medicines, especially those with Rasayana properties as in the current study, may fulfill the requirement of cartilage repair and chondroprotection. This proposition and several other hypotheses are being carefully tested in the NMITLI project before Ayurveda can address some of the unmet needs in modern medicine [35]. We have recently completed an appropriately powered and designed drug trial study of Ayurvedic “C” formulation in the long-term treatment of OA knees which also includes surrogate measures of cartilage damage and chondroprotection (not yet published).

In conclusion, we have demonstrated a better efficacy and safety profile of a standardized Ayurvedic formulation “C” in the symptomatic treatment of OA knees using a fast track multiple interventional arm exploratory drug trial controlled for placebo and glucosamine. The latter was a preliminary phase of a national project (NMITLI) and formulation “C” was chosen for further development and validation. Some of the logistic and socioeconomic issues connected with trials on traditional medicine, especially in the Indian scenario, are highlighted. The importance of using validated health assessment instruments appropriate for regional use such as Indian version of WOMAC is also emphasized. We also recommend a multidisciplinary integrated approach to validate traditional medicines.

##  Contributors to Paper

The clinical strategy and current protocol was designed by A. Chopra and finalized with important inputs from M. Saluja (coordinator), A. Raut (Ayurveda), G. Tillu (Ayurveda) and A. Venugopalan (laboratory). The principal investigators (A. Chopra, G. Narsimulu, L. Bichile, and A. Raut) vouch for the integrity and correctness of the clinical trial data. The statistical plan, analysis, and results were provided by S. Sarmukaddam. The final manuscript was prepared by A. Chopra, M. Saluja, G. Tillu and B. Patwardhan and confirmed for correctness by all the coauthors. As Chair of NMITLI arthritis project, BP was responsible for the NMITLI concept, rationale, and quality of test materials.

##  Funding

This work was fully funded and supported by NMITLI Cell, Council of Scientific and Industrial Research (CSIR), Government of India.

## Supplementary Material

Adverse Event episodes in patients by treatment groups.

## Figures and Tables

**Table 1:** Selected description of medicinal plants (Ayurvedic) and their extracts used to treat symptomatic knee osteoarthritis in a randomized controlled exploratory study.

Common name	Botanical name (Family)	Part used (Extract type)	Voucher specimen number*	Phytomarker standardization by HPLC
*Shunthi*	*Zingiber officinale Roscoe.* (Zingiberaceae)	Dried Rhizomes (Powder)	*R*-035	Total Gingerols
*Guduchi*	*Tinospora cordifolia* Miers. (Menispermaceae)	Dried stem (Aqueous extract)	*R*-034	Tinosporosides
*Amalaki*	*Emblica officinalis* Gaertn (Euphorbiaceae)	Dried fruits (Aqueous extract)	*F*-033	Total tannins Galic acid
*Ashwagandha*	*Withania somnifera *Dunal (Solanaceae)	Dried roots (Aqueous extract)	*R*-033	Total Withanolides
*Gokshur*	*Tribulus terrestris* Linn. (Zygophyllaceae)	Dried fruits (Aqueous extract)	*F*-030	Total Saponins

*Voucher specimen deposited in Agharkar Herbarium at Maharashtra Association for the cultivation of science (AHMA), Pune.

**Table 2:** Drug codes of Ayurvedic formulations along with the daily dosage regimen (of plant extracts) and comparators (placebo and glucosamine) used to treat symptomatic knee osteoarthritis in a randomized controlled exploratory study.

Number	Ingredients with daily dose	Code
1	*Shunthi *1000 mg + *Guduchi *220 mg	D
2	*Shunthi *1000 mg + *Guduchi * 220 mg + Amalaki 500 mg	C
3	*Shunthi* 1000 mg + *Guduchi *220 mg + *Ashwagandha *600 mg	E
4	*Shunthi* 1000 mg* + Guduchi *220 mg* + Gokshur *216 mg	A
5	*Shunthi *1000 mg + *Guduchi* 220 mg + *Ashwagandha* 600 mg + *Gokshur *216 mg	B
6	Placebo (Maize starch)	F
7	Glucosamine sulphate	G

**Table 3:** Demographic and baseline variables of patients (*n* = 245) by treatment groups: Ayurvedic arms code (A–E), glucosamine (GLU), and placebo (PLB).

	A	B	C	D	E	PLB	GLU
Number	34	33	35	33	34	34	33
Age (years, mean ± SD)	57.5 ± 7.8	56.6 ± 9.4	56.8 ± 8.1	56.2 ± 9.2	56.2 ± 9.5	54 ± 7.7	54.2 ± 8.1
Weight (Kg ± SD)	63.8 ± 11.2	66 ± 13.6	64.3 ± 13.9	63.5 ±10.5	63.8 ± 10.1	65.6 ± 11.0	65.6 ± 11.8
BMI (Mean, ±SD)	28.3 ± 4.6	28.8 ± 7.5	26.9 ± 4.4	27.7 ± 4.8	27.5 ± 4.5	28.1 ± 5.3	27.6 ± 4.4
Duration of disease (years)	3.9	6.8	4.8	5.1	5.4	4.7	5.9
Active-pain VAS	6.5 (1.2)	6.2 (1.2)	6.3 (1.4)	6.2 (1.2)	6.4 (1.3)	6.4 (1.5)	6.5 (1.4)
W-PAINpPain Pain	9.0 (3.0)	8.3 (3.5)	8.0 (3.0)	8.2 (4.0)	9.2 (3.0)	9.0 (3.7)	8.0 (3.4)
W-DIFF	27 (11.7)	27 (12.2)	25.5 (8.9)	25.0 (12.9)	27.0 (12.3)	28.2 (11.7)	26.0 (10.4)

SD: standard deviation; BMI: body mass index; VAS: visual analogue scale; W: WOMAC questionnaire for functional disability index for knee and hip (see text for details); DIFF: difficulty.

**Table 4:** Mean change (percent) in primary efficacy variables, pain visual analogue scale (VAS) and WOMAC (W) index score, and oral tablet paracetamol (PARA) consumption from baseline at 16 week completion end point in patients (*n* = 245) by treatment groups: Ayurvedic arms code (A–E), glucosamine (GLU), and placebo (PLB); intent to treat analysis.

Code	ActivePAINVAS	W-PAIN	W-DIFF	PARA 7	PARA-T
A (*n* = 31)	0.9 (13.5)	2.3 (17.2)	5.0 (16.5)	1.5 (0.6)	2.1 (0.8)
B (*n* = 33)	1.6 (24)	2.2 (11.6)	6.9 (22.0)	1.3 (0.8)	1.6 (0.9)
C (*n* = 30)	1.8 (26)	2.5 (26.7)	5.3 (17.6)	0.9 (0.8)*	1.3 (1.0)*
D (*n* = 29)	1.2 (18.7)	2.3 (12.9)	5.0 (12.4)	1 (0.9)	1.3 (1)
E (*n* = 29)	1.3 (19.3)	2.5 (25.9)	5.0 (12.1)	1.5 (0.8)	1.9 (0.9)
GLU (*n* = 29)	1.43 (18.4)	1.5 (14.2)	4.4 (14.2)	1.4 (0.8)	1.8 (1.1)
PLB (*n* = 27)	1.38 (18.7)	2.9 (28.2)	7.5 (24.3)	1.2 (0.9)	1.5 (1)

N: number;*:*P* < .05; W: WOMAC questionnaire for functional disability index for knee and hip (see text for details); DIFF: difficulty; PARA 7: mean number of oral paracetamol tablets (each 500 mg) consumed daily during the last four weeks of study as a rescue analgesic, *F* value (ANOVA) = 2.3 (*P* < .05); PARA T: mean number of oral paracetamol tablets (each 500 mg) consumed daily during the study period as a rescue analgesic, F(ANOVA) = 2.6 (*P* < .05).

**Table 5:** Comparison of the change in the knee status of patients of symptomatic knee osteoarthritis treated with Ayurvedic “C” formulation (found to be most efficacious), placebo, and glucosamine at completion in a seven arm (5 Ayurvedic formulations) randomized controlled exploratory drug trial using a RIDIT (relative to identified distribution) analysis.

Comparison pair	Mean RIDIT	“Z”	*P*
C versus placebo	0.652	2.185	.029
C versus glucosamine	0.618	1.692	.09

Knee status on study completion scored by patient using categorical outcome (see text for details).
