# Supplementary material for: A Randomized Controlled Exploratory Evaluation of Standardized Ayurvedic Formulations in Symptomatic Osteoarthritis Knees: A Government of India NMITLI Project
Source: Evid Based Complement Alternat Med. 2010 Oct 11;2011:724291. doi: 10.1155/2011/724291 (PMC2964493; doi:10.1155/2011/724291)
Supplement: Supplementary file 1 — Adverse Event episodes in patients by treatment groups. [file 724291.f1.pdf]

Supplementary Data: Adverse Event episodes in patients by treatment groups

(This data is presented as histograms as Figure 4.)

Number (percent) of Adverse Event episodes in patients (n=245) by treatment groups- Ayurvedic arms code A-E, glucosamine (GLU) and placebo (PLB)

Adverse Event	A (n=35)	B (n=35)	C (n= 35)	D (n=35)	E (n=35)	GLU (n=35)	PLB (n=35)
Epigastric discomfort	6 (17.1)	13 (37.1)	4 (11.4)	3 (8.6)	11 (31.4)	7 (20)	9 (25.7)
Diffuse Pain abdomen	4 (11.4)	4 (11.4)	2 (5.7)	2 (5.7)	2 (5.7)	4 (11.4)	4 (11.4)
Anorexia	-	1(2.9)	1 (2.9)	2 (5.7)	1 (2.9)	1 (2.9)	1 (2.9)
Nausea	6 (17.1)	4 (11.4)	5 (14.2)	3 (8.6)	5 (14.2)	6 (17.1)	4 (11.4)
Vomiting	1 (2.9)	1 (2.9)	-	-	3 (8.6)	1 (2.9)	-
Diarrhoea	3 (8.6)	1 (2.9)	1 (2.9)	-	2 (5.7)	1 (2.9)	2 (5.7)
Constipation	1 (2.9)	3 (8.6)	1 (2.9)	-	-	2 (5.7)	3 (8.6)
Oral Ulcers	1 (2.9)	1 (2.9)	-	-	1 (2.9)	-	1 (2.9)
Skin Rash & Itching	-	3 (8.6)	4 (11.4)	3 (8.6)	3 (8.6)	1 (2.9)	6 (17.1)
